# Ischemic Cardiomyopathy and Cerebral Infarction in a Young Patient Associated with Khat Chewing

**DOI:** 10.1155/2015/893176

**Published:** 2015-02-26

**Authors:** T. J. Meulman, J. Bakker, E. J. van den Bos

**Affiliations:** ^1^Department of Radiology, Albert Schweitzer Ziekenhuis, Postbus 444, Dordrecht, 3300 AK Dordrecht, Netherlands; ^2^Department of Cardiology, Albert Schweitzer Ziekenhuis, Postbus 444, Dordrecht, 3300 AK Dordrecht, Netherlands

## Abstract

Khat is a stimulating agent used by many people in the Horn of Africa and the Arabian peninsula. Khat chewing is a known cardiovascular risk factor and is thought to cause vasoconstriction, systemic hypertension, and thrombogenicity. A 33-year-old Somalian man initially presented with loss of neurological function of the left arm, hazy vision, and headache. He smokes tobacco and chews two bundles of khat a week for more than 10 years. His ECG on admission showed a Q wave in V1 and V2 and 2 mm ST-elevations in V1, V2, and V3 and a terminal negative T wave in I, aVL, V2, V3, and V4, consistent with a recent, evolving anterior infarction. A noncontrast enhanced CT of the brain showed ischemia in the right middle cerebral artery vascular territory. An MRI showed recent ischemia in the vascular territory of the posterior division of the right middle cerebral artery. Coronary angiography showed a 70% stenosis with haziness of the proximal left anterior descending artery. Diagnostic tests and imaging are consistent with recent myocardial infarction in the LAD vascular territory because of coronary spasm and cerebral infarction in the middle cerebral artery vascular territory probably related to khat chewing.

## 1. Case Presentation

**Figure 1:**
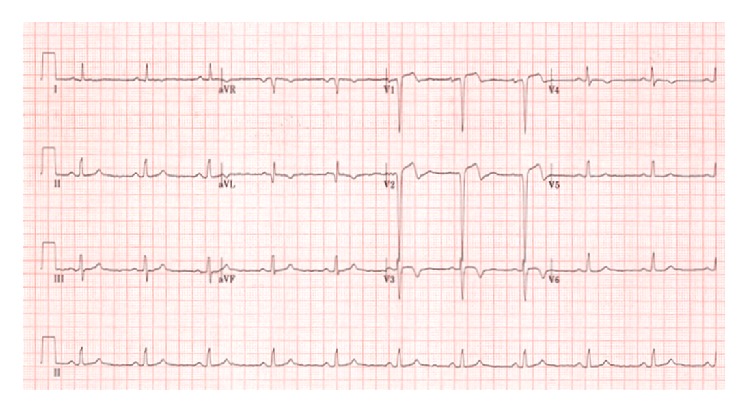
ECG. The ECG on admission showed a Q wave in V1 and V2 and 2 mm ST-elevations in V1, V2, and V3 and a terminal negative T wave in I, aVL, V2, V3, and V4, consistent with anteroseptal infarction.

A 33-year-old Somalian man initially presented with loss of neurological function of the left arm, hazy vision, and headache. A few weeks prior to this episode he had had an episode during which he experienced chest pain, nausea, and vomiting. Our patient does not drink alcohol. He smokes tobacco and chews two bundles of khat a week for more than 10 years. No other risk factors for atherosclerosis at young age were present. The blood levels of cholesterol and triglycerides were within normal range. Family history was negative for inherited thrombophilia. His ECG on admission showed a Q wave in V1 and V2 and 2 mm ST-elevations in V1, V2, and V3 and a terminal negative T wave in I, aVL, V2, V3, and V4, consistent with a recent, evolving anterior infarction (Figure 1). On admission the highly sensitive troponin T was 18 ng/L (normal value < 14 ng/L). The transthoracic echocardiogram showed a poor left ventricular function with akinesia of the anterior wall and left ventricular dilatation. No thrombus was seen.

The noncontrast enhanced CT of the brain showed ischemia in the right middle cerebral artery vascular territory. The CTA of the carotids showed no stenosis or venous thrombosis.

**Figure 2:**
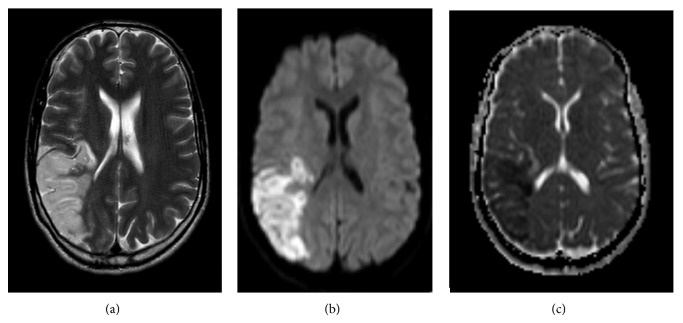
MRI of the brain. (a) T2-weighted image, (b) diffusion weighted image, and (c) apparent diffusion coefficient (ADC) map, showing high signal intensity on the T2-weighted and diffusion weighted image and low signal intensity on the ADC map in the vascular territory of the posterior division of the right middle cerebral artery, consistent with recent ischemia.

An MRI of the brain was performed to rule out vasculitis or other vascular malformations. The MRI showed recent ischemia in the vascular territory of the posterior division of the right middle cerebral artery (Figures 2(a)–2(c)).

**Figure 3:**
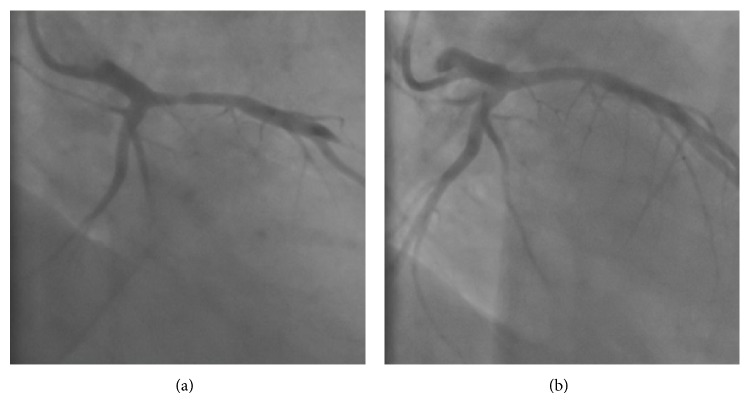
Coronary angiogram. (a) Coronary angiogram showing a 70% stenosis, haziness, and spasm of the left anterior descending artery (LAD). (b) The stenosis disappeared after intracoronary nitroglycerine injection.

Coronary angiography showed a 70% stenosis with haziness of the proximal left anterior descending artery (LAD) (Figure 3(a)), which disappeared after intracoronary nitroglycerine injection (Figure 3(b)). The other coronary arteries were normal.

**Figure 4:**
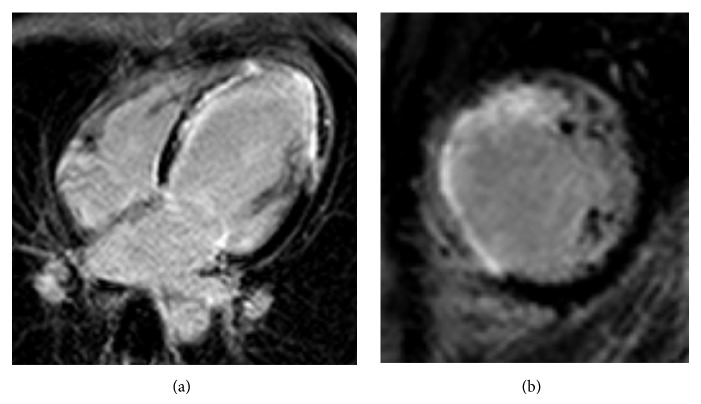
Cardiac MRI. (a) Four-chamber view, obtained 15 minutes after intravenous gadolinium injection. The anterolateral segment shows delayed enhancement in less than 50% of the myocardial thickness. (b) Short-axis view, obtained 15 minutes after intravenous gadolinium injection, showing transmural delayed enhancement of the midcavitary septal segment.

MRI of the heart showed a poor left ventricular function, with a calculated ejection fraction of 29%. The left ventricle was dilated, with an end diastolic left ventricular diameter of 63 mm (normal value < 55 mm). There was thinning of the myocardium in the apex and the midcavitary anteroseptal, anterior, and anterolateral segments. There was hypo- to akinesia of these areas with subendocardial and transmural delayed enhancement (Figures 4(a) and 4(b)) in these regions. No thrombi were seen. The signs on the MRI are consistent with infarction in the vascular territory of the LAD.

Screening for thrombophilia (lupus anticoagulants, cardiolipin antibodies, factor V Leiden, and antithrombin III deficiency) was negative. A presumptive diagnosis was made of khat induced coronary spasm with myocardial infarction and khat induced cerebral infarction. Due to hypotension treatment with calcium blocking agents was not possible. He was advised to refrain from khat use. Furthermore he underwent a percutaneous coronary intervention of the proximal LAD with stent placement to prevent stenosis in case of possible future coronary spasm. His left ventricular function remained poor, which was a reason for ICD implantation for primary prevention. During admission all neurological complaints resolved.

## 2. Discussion

Khat (*Catha edulis*) is a stimulating agent used by many people in the Horn of Africa and the Arabian Peninsula. The chewing of khat leaves is a social custom in these areas. Immigrants from these areas have spread the custom to other parts of the world. Khat contains a monoamine alkaloid called cathinone, which is said to cause euphoria, alertness, and central nervous system stimulation [1].

Khat chewing is a known cardiovascular risk factor and is thought to cause vasoconstriction, systemic hypertension, and thrombogenicity [2]. Links have been proposed between khat chewing and the incidence of myocardial infarction, dilated cardiomyopathy, vascular disease such as hypertension, cerebrovascular ischaemia and thromboembolism, diabetes, sexual dysfunction, duodenal ulcer, and hepatitis [1]. Ali et al. found that khat chewers had higher risk of death, recurrent myocardial ischemia, cardiogenic shock, ventricular arrhythmia, and stroke compared with nonkhat chewers and khat chewing was found to be an independent risk factor of death and for recurrent ischemia, heart failure, and stroke [3].

Patients with acute coronary syndrome related to khat use typically present later than non-khat related acute coronary syndrome because of the analgesic effect of khat [2].

Earlier case reports have been presented with patients suffering from either cardiovascular complications or cerebrovascular complications of khat chewing [4]. As far as we know only one case report presented a patient with a combination of cardiovascular and cerebrovascular complications related to khat use [5].

## 3. Conclusion

A young Somalian patient presented with severe myocardial infarction in the LAD vascular territory because of coronary spasm and cerebral infarction in the middle cerebral artery vascular territory. Our patient presented with both cardiac and cerebrovascular complications, both probably related to a combination of smoking and khat chewing. In both cases this was confirmed by MRI with typical imaging characteristics of ischemic cardiomyopathy and cerebral infarction.

## Abbreviations

ECG: Electrocardiography
CT: Computed tomography
CTA: Computed tomography angiography
MRI: Magnetic resonance imaging
LAD: Left anterior descending artery
ICD: Implantable cardioverter defibrillator
ADC: Apparent diffusion coefficient.
